# 

**Published:** 1912-05-25

**Authors:** 


### New Tropical Diseases Bureau.

The announcement by the Colonial Office that the Sleeping Sickness Bureau will in future be known as the Tropical Diseases Bureau marks an important epoch in the history of tropical medicine. For a long time this Bureau, housed by the Royal Society, has extended its sphere of action beyond the field of the trypanosome, and it is extremely opportune that there should be taken under the wing of the Government a Commission which will collect and publish the discoveries that are being made in tropical medicine all over the world. That the control of the Bureau will be in the hands of Sir Patrick Manson, Sir David Bruce, Sir W. Leishman, and Sir J. Rose Bradford is guarantee that both genius and vigour will accompany the new departure.

The new Bureau will be of great value to the London School of Tropical Medicine. The close association which has ever existed between the school and the Colonial Office will afford much mutual help both to the Bureau and the school. To the appeal on behalf of tropical medicine set forth by Mr. Lewis Harcourt at the Mansion House in February, £4,500 has already been received. This will be devoted to the enlargement of the school, and Mr. Austen Chamberlain has consented to accept the post of chairman of a committee which is being formed to continue the appeal, not only to extend the laboratories, but to increase the endowment fund, to provide a nursing home for Government employees and officials generally of limited resources who may be suffering from tropical diseases, and also for research. Sir William Bennett's allocation of Lord Wandsworth's bequest is referred to elsewhere in this issue.

### OUR BUREAU OF INFORMATION.

### Rules for Correspondents.

1. Every letter, on whatever subject, must be accompanied by the coupon to be cut from the back cover (inside page) of The Hospital, current issue, and must contain the name and address of the correspondent with pseudonym for publication if desired.

2. Replies by post cannot be given, save under exceptional circumstances, at the Editor's discretion.

3. Letters from Approved Homes in reply to special needs published in the Bureau should state terms and full particulars, and be sent prepaid under cover to the Editor of the Bureau with coupon and name enclosed for identification.

4. Proprietors of homes which have not yet been entered on the List of Approved Homes, but have spare accommodation likely to suit special needs, are invited to write for an application form for registration. The fee for registration is 5s.

5. Special needs of every description relating to those who are sick or in difficulties are made known in these columns free of charge, but applications relating to business or employment from correspondents in an independent position must be accompanied by a postal order for 5s.

6. All communications to be addressed to The Editor of The Hospital Bureau of Information, 28 Southampton Street, Strand, London, W.C.

### Hospital Management and Training.

### Invalid Cookery Classes in Small General Hospitals.

Miss Bamber.—The system adopted depends on whether one of the staff is qualified to give this instruction. In this case, which is greatly to be preferred, cookery courses are planned to fit in with other lectures, and the matron takes care to arrange that no probationer misses an opportunity of studying this necessary subject during her time in the hospital. It is more satisfactory to give the training to probationers in their first year, as it makes a welcome relief to nursing duties, and as they are then of least use in the wards they can better be spared than later. Should there, however, be no member of the staff qualified to give a course on cookery it is necessary to engage a lecturer from outside, either from the nearest town or through the county council, which in some counties employs teachers and will undertake classes wherever formed at low fees. In this case a course would be given once a year, and the matron would set free as many nurses as possible to take advantage of the lectures, though it could hardly be possible for her to insure that all her nurses were able to go through the course.

It is essential to remember that "Invalid Cookery" is not merely practice in working out certain recipes. It should be taken in a far wider sense as instruction in the component parts of ordinary articles of diet, with their respective value in building up the body and maintaining energy. The pupils should be led to understand in what way the various substances used as food are modified by cooking, and should attack the problem of invalid cookery by learning the processes which best prepare food for assimilation by persons with impaired digestive apparatus. The art of preparing food for those in normal health does not differ materially from that of preparing it for the sick, and if the former be well taught, the last two or three lessons will be found sufficient for demonstrations on the preparation of dishes for invalids.

### Training for Male Nurse.

"E. W. R.," Leicester.—The post of dresser is held only by medical students. Write to the Matron, National Hospital for the Paralysed and Epileptic, Queen Square, Bloomsbury, London, for particulars of the course of training in this institution for male nurses. As you are interested in the medical and surgical aspects of nursing you would probably find the work of the Royal Army Medical Corps congenial. The recruiting officer in your town would give you full particulars regarding the pay and conditions of service. Most of the large Poor-Law infirmaries employ male attendants, and your present certificate would probably suffice to qualify you for such posts. You might apply for any post of this kind you see advertised, if you do not wish to undergo regular training, as in these institutions they would expect to give the male attendants a certain amount of preliminary instruction in their duties.

### Hospital Finance.

### The Income of Voluntary Hospitals.

"Vigilans."—It is remarkable that your erroneous impression that the hospitals have suffered in income by the establishment of the King's Fund is so widespread among those who ought to know better. The figures you want are as follows:—In 1896 the income of the Metropolitan hospitals and dispensaries, exclusive of legacies, amounted to £761,970; in 1908 it amounted to £1,149,363. During the same period the income of the great collecting funds had increased from £68,000 in the year before the King's Fund began its operations to £234,000, a change which has brought an additional sum of over £200,000 a year into the coffers of the hospitals. It is evident, therefore, that contributors to particular hospitals have been stimulated by the interest aroused through the efforts made on behalf of the King's Fund, and that all have benefited individually as well as collectively. We refer you for further particulars on the subject to Burdett's "Hospitals and Charities," the new edition of which is announced.

### Needs and Helpers.

### Home of Rest for Gentlewomen.

Central Bureau for the Employment of Women.—We shall be happy to forward to the right quarter applications from any candidates you recommend for admission to the Home of Rest, if they will kindly comply with our rules and send them under cover to the Editor of The Bureau with coupon of the current issue of The Hospital. It is exactly such centres as your own as are likely to meet with the cases most in need of this timely benefit, and we are very glad to co-operate with your admirable society.

"Miss F. M. Smith."—We are sending on the application for Miss R. to the right quarter. Please remember coupon in sending letters.

### Home for Small Payment with Services.

"Mrs. I.," Herne Bay.—Have received your very kind letter and forwarded it to Mrs. D. I am glad to know from her that she accepts your kind offer gratefully, and I trust terms may be arranged on a mutually satisfactory basis.

### Letter Box.

"Mrs. R.," Barking.—Regret we are unable to forward letters until you have complied with Rule 4.

"Miss I."—Have forwarded your letter and have recommended "Francis" to accept your advantageous proposal.

Readers of The Hospital are invited to bring these columns under the notice of their friends who are engaged in philanthropic work of any description. We want the co-operation of district nurses, district visitors, and health visitors. We want the clergy to send us particulars of cases which need institution treatment. All who are trying to help their neighbours are faced with the problem of fitting the remedy to the need. It is this problem we aim at solving.

*To the Editor of* The Hospital.

### Appointments.

Dr. W. H. Maxwell Telling, M.D., B.S., M.R.C.P., M.R.C.S., has been elected honorary physician to the Leeds General Infirmary.

Dr. J. Russell Orchard, assistant resident medical officer of St. Mary's Hospital, Plaistow, London, has been appointed house surgeon to the Torbay Hospital.

Mr. H. G. R. Jamieson, M.B., of the Royal Hampshire County Hospital, Winchester, has been appointed house surgeon at the Herefordshire General Hospital for a period of six months, at the end of which time he will be eligible for re-election.

Mr. F. F. Burghard, M.S., F.R.C.S., and Mr. C. H. Fagge, M.S., F.R.C.S., have been appointed honorary consulting physicians to the Surbiton Cottage Hospital, and Dr. W. H. Jewell, M.D., B.S., has been appointed honorary consulting ophthalmic surgeon.

The following appointments of honorary medical officers at the Salford Royal Hospital have been made:—Frank E. Tylecote, M.D., Ch.B., D.P.H. (Victoria), M.R.C.P. (London), an honorary physician to fill the vacancy caused by the death of the late Dr. Dixon Mann, and Hugh T. Ashby, B.A., M.B., B.C. (Cantab.), M.R.C.P. (London), an honorary assistant physician. It has been decided to name the new medical ward, one of Dr. Mann's, the "Dixon Mann Ward," to erect a commemorative tablet in the hall, and to reproduce Dr. Mann's portrait.

Mr. E. Musgrave Woodman, M.B. (Lond.), M.S. (Lond.), F.R.C.S. (Eng.), of St. Bartholomew's Hospital, London, has been appointed assistant surgeon to the General Hospital, Birmingham, for a term of three years, when he will be eligible for re-election. Mr. Woodman has been honorary surgeon to the Marylebone General Dispensary, chief assistant to surgical out-patients at St. Bartholomew's Hospital, surgical registrar to the cancer wards of Middlesex Hospital, house surgeon at St. Bartholomew's Hospital, and assistant medical superintendent at St. Pancras Infirmary.

The following appointments at the Manchester Royal Infirmary have been confirmed:—To be house physicians: Mr. F. S. Bedale, M.A. (Cantab.), M.R.C.S., L.R.C.P., Mr. Roger Stewart, M.R.C.S., L.R.C.P., and Dr. James M. Scott, M.B., Ch.B. (Edin.). To be senior house surgeons: Dr. S. B. Radley, M.B., Ch.B. (Vict.), and Dr. W. H. Kauntze, M.B., Ch.B. (Vict.). To be junior house surgeons: Dr. N. Duggan, M.B., Ch.B. (Vict.), M.R.C.S., L.R.C.P., and Dr. N. Matthews, M.B., Ch.B. (Vict). In addition Dr. G. K. Thompson, M.B., Ch.B. (Vict.), has been appointed house surgeon to special department, and Dr. C. E. Lea, M.D. (Vict.), to be medical registrar.

A Whitsun Holiday.—By a fortunate coincidence of dates a luxurious fortnight's sea-going Whitsuntide holiday can be secured by the sailing from London on May 24 of the Orient Line mail steamer. The outward part of the trip is made by one of the company's new 12,000-ton vessels, and the return voyage is by the largest and latest addition to the Orient Line, the "Orama" (12,928 tons). The accommodation on both vessels includes *cabines de luxe,* with private sitting-room and bath-room, bedstead cabins, and single-berth rooms. Both the vessels are fitted with wireless telegraphy. Gibraltar is reached on May 28, and six days may be spent in visiting the Moorish cities of Spain or Africa, or in a short riding tour in Southern Spain. Ferry steamers connect Gibraltar with the railway terminus at Algeciras, whence trains run for Ronda and Bobadilla, the junction for Granada and Seville. The fare for the fifteen days' trip ranges from £16 saloon and £11 second saloon.

